# Prevalence and predictors of aortic dilation as a novel cardiovascular complication in children with end-stage renal disease 

**DOI:** 10.5414/CN108489

**Published:** 2015-03-27

**Authors:** Ahmad Kaddourah, Susan Uthup, Peace Madueme, Matthew O’Rourke, David K. Hooper, Michael D. Taylor, Steven D. Colan, John L. Jefferies, Marepalli B. Rao, Jens Goebel

**Affiliations:** 1 Nephrology Division,; 2 The Heart Institute, Cincinnati Children’s Hospital Medical Center, Cincinnati, OH,; 3 Cardiology Division, Boston Children’s Hospital, Boston, MA, and; 4 Biostatistics & Epidemiology Division, Department of Environmental Health, University of Cincinnati, Cincinnati, OH, USA

**Keywords:** aortic dilation, end-stage renal disease, cardiovascular disease, kidney transplant

## Abstract

Background: Cardiovascular disease is the leading cause of death in children with end-stage renal disease (ESRD). Isolated aortic dilation (AD) is rare in children. We aimed to determine the prevalence and the risk factors for AD in children with ESRD. Methods and study design: We reviewed records of all ESRD patients followed at our institution from January 2007 to October 2012. AD was defined as Z-score > 2 in the dimension of at least one of the following echocardiographic aortic parameters: annulus, root at the sinus, sino-tubular junction, ,or ascending aorta. Results: The records of 78 patients on dialysis and 19 kidney transplant recipients were available. 30 patients (30.9%) had AD. Multivariate analysis revealed independent associations of AD with body mass index (BMI) Z-score (OR = 0.52, 95% confidence interval (CI): 0.35 – 0.78) and ESRD secondary to glomerular disease (OR = 4.58, 95% CI: 1.45 – 14.46). We developed a classification and regression tree (CART) model to identify patients at low vs. high AD risk. Our model classified 62 patients of the cohort (64%) to be high- or low-risk, with a positive predictive value of 89% and a negative predictive value of 100%. Conclusion: Our data suggest that AD, as a possible marker of aortopathy and early aneurysm formation, is a novel and prevalent cardiovascular complication in ESRD children. Glomerular disease and low BMI Z-score appear to be potent predictors. CART modeling helps identify high-risk children, potentially guiding decisions regarding targeted echocardiographic evaluations.

## List of abbreviations 

AD = aortic dilation; BMI = body mass index; BP = blood pressure; CART = classification and regression tree; CCHMC = Cincinnati Children’s Hospital Medical Center; CKD = chronic kidney disease; CVD = cardiovascular disease; DBP = diastolic blood pressure; EDW = estimated dry weight; ESRD = end-stage renal disease; FSGS = focal segmental glomerulosclerosis; IDWG = interdialytic weight gain; iPTH = intact parathyroid hormone; SBP = systolic blood pressure; PD = peritoneal dialysis; UF = ultrafiltration 

## Background 

Aortopathy, defined as pathological dilation of the aortic root and/or the ascending aorta is extremely rare in healthy individuals but can have devastating outcomes, such as aortic dissection and aneurysm formation with subsequent rupture [1]. While cardiovascular disease (CVD) manifesting as advanced atherosclerosis, abnormal cardiac remodeling, and impairment of systolic and diastolic functions has been extensively studied in patients with chronic kidney disease (CKD) and end-stage renal disease (ESRD) [2], structural abnormalities like aortic dilation (AD) have rarely been documented in this population. The survival of children with ESRD continues to be undesirably low, and the most likely cause of death remains CVD [3, 4]. Our institutional practice is annual echocardiography in all patients with ESRD, and we noted that aortic dilation was a frequently reported finding, especially in patients with glomerular disease. We therefore undertook a systematic, single-center review of the prevalence of and risk factors for AD in our ESRD population and tested the hypothesis that the prevalence of AD is higher among children with ESRD secondary to glomerular disease as compared to those with ESRD due to nonglomerular disorders. 

## Study population and methods 

With approval by Cincinnati Children’s Hospital Medical Center (CCHMC)’s institutional review board, we retrospectively and cross-sectionally studied all patients who received ESRD care at our institution between January 2007 and October 2012. ESRD was defined as undergoing chronic hemodialysis (HD) or peritoneal dialysis (PD) or having received a kidney transplant. Additional specific inclusion criteria were age below 23 years, absence of congenital or structural heart disease, including bicuspid aortic valve, and absence of disorders known to be associated with a high incidence of aortic diseases, such as Marfan, Turner, Loeys-Dietz, or Ehlers-Danlos syndromes [5]. 

## Predictors 

Demographic and clinical data were extracted from each patient’s medical records. The clinical parameters of interest were collected closest to the time of echocardiography and are shown in Table 1. Body mass index (BMI) was determined using the Quetelet index: BMI (kg/m^2^) = weight (kg)/height (m) [2]. Systolic and diastolic blood pressure (BP) readings during the month prior to the date of echocardiography were extracted from medical records when available. BP status was defined based on the Fourth Report on the diagnosis, evaluation, and treatment of high BP in children and adolescents [6]. BP was adjusted for body size for direct comparison across all age groups, and the calculated mean BP values were divided by the age-, gender-, and height-specific 95^th^ percentiles for both systolic (SBP) and diastolic BP (DBP) to determine the respective BP indices for each subject. SBP or DBP indices > 1.0 were defined as uncontrolled hypertension [7]. Dyslipidemia was defined on the basis of the kidney disease outcomes quality initiative guidelines [8]. 

The dialysis parameters of interdialytic weight gain (IDWG), ultrafiltration (UF) volume, and Kt/V were determined from the dialysis records for the month prior to echocardiography. For children on HD, the collected data included pre- and posttreatment weights, estimated dry weight (EDW), and average UF for each of 12 consecutive HD sessions. Average IDWG was calculated from the difference between mean pretreatment weights and mean posttreatment weights from the preceding HD sessions. Average UF was calculated from the difference between mean pretreatment weights and mean posttreatment weights. Average excess weight was calculated from the difference between mean posttreatment weight and mean EDW. Normalized values for IDWG, UF, and excess weight were also calculated for each subject by dividing their mean values by the subject’s EDW and then multiplying by 100 [7]. Children on PD received nightly treatments of continuously cycling PD. Average UF on PD was divided by body surface area to yield corrected UF for analysis. Data on dialysis adequacy, as estimated by double-pool Kt/V values for the month prior to echocardiography, were also collected. 

### Echocardiograms 

Only one echocardiogram study was analyzed for every enrolled patient. When multiple echocardiograms were available, we chose: 1) the most recent study for patients on dialysis who had not received a kidney transplant, 2) the latest pretransplant study on dialysis for patients who subsequently underwent kidney transplantation, and 3) the first posttransplant study for patients with no available pretransplantation study. With this strategy, we aimed to capture the biggest possible effect of ESRD on aortic pathology and to simultaneously avoid as much as possible any potential improvements in aortic dilation after transplantation. Echocardiograms were retrospectively identified and had been obtained while at rest using a Vivid 7 GE ultrasound imaging system (Milwaukee, WI, USA) or a Philips iE33 x MATRIX ultrasound system (Andover, MA, USA). Testing was performed by pediatric registered sonographers. Images were obtained in standard views according to the American Society of Echocardiography’s guidelines [9, 10]. Echocardiographic data were obtained in the parasternal long axis view utilizing an inner edge to inner edge technique during systole and included aortic measurements at the sinus of Valsalva, annulus, sino-tubular junction, and ascending aorta. Inter- and intraobserver variability for these measurements in our laboratory is less than or equal to 5%. The Z scores of the aortic dimensions were calculated using the regression models of Boston Children’s Hospital echocardiography laboratory [11]. In these models, BSA (body surface area) is the only variable used in the equation and was calculated using Haycock formula [12]. AD was recognized when the Z-score of at least one of the aortic dimensions was greater than 2 [13]. 

Study data were collected and managed using REDCap^®^ [14]. REDCap^®^ is a secure web-based application designed to support data capture for research studies. 

## Statistical analysis 

Categorical data are presented as counts and percentages and were analyzed with Fisher’s exact test. Continuous data are presented as means and standard deviations (SDs) and analyzed with Student’s t-test. p-values less than 0.05 were considered statistically significant. Two multivariate logistic regression models were created to identify predictors of AD. Model 1 includes the variables of p-values ≤ 0.15 in the univariate analysis. Model 2 includes the clinical variables that are known to be associated with CVD in addition to the variables included in model 1.We developed risk prediction models using recursive partitioning (classification and regression tree, CART), for the presence or absence of AD. CART creates nonparametric discriminating trees by dividing the cohort into subgroups representing high or low risk of AD based on available clinical variables. 

The statistical analyses were conducted using version 2.13.0 of the R statistical package, SAS version 9.3 (SAS Institute Inc., Cary, NC, USA). 

## Results 

### Demographics 

Of the 133 patients with ESRD during the study period, 130 met the inclusion criteria, although 33 of them only had incomplete echocardiographic records, leaving data from 97 children to be analyzed. The mean age of the population was 11.5 (6.5) (range 0.2 – 22.7) years. The majority of subjects were male (56.7%), Caucasian (77.3%) and had nonglomerular disease (55.7%). 19 patients had no pretransplant echocardiograms available and were thus studied posttransplant. From the remaining 78 patients, echocardiograms obtained on chronic dialysis were available and thus included. 34 of these patients had been on HD only, 41 on PD only, and 3 had switched modalities. Even though 80 subjects (82.4%) were prescribed antihypertensive medications, hypertension (defined as SBP or DBP indices > 1), was still present in 66 (82.5%). 

### Incidence of aortic dilation and echocardiographic details 

AD was found in 30 of 97 (30.9%) patients. The mean age of the AD group was 12.3 (6.1) years. The mean values of aortic dimensions in the patients with and without AD are shown in Table 1. The aortic dimensions showed significant differences between the two groups at all the measured levels, but the difference was most apparent at the sinus. When considering a Z-score of > 2 as dilated, 25 of 30 (83.3%) patients had dilation at the sinus, 13 of 30 (43.3%) had it at the sino-tubular junction, and 22 of 30 (73.3%) at the ascending aorta. Only 1 child had a dilated annulus. Mild aortic regurgitation was observed in 10 patients in the AD group (30%), and all of them except 1 had a dilated ascending aorta. 

### Univariate Analyses (Table 1) 

Both groups were comparable with regards to age and sex, but children with AD had a lower BMI compared to children without AD: 18.4 (3.7) vs. 23.3 (7.15) kg/m^2^ (p = 0.001). This difference was still significant when we compared the BMI Z-scores (–0.7 (1.7) vs. 0.9 (1.3), p < 0.0001). Interestingly, 7 (23.3%) children with AD were malnourished, defined as BMI Z-score < –2, compared to none in the group without AD. While more than half (54/97, 58.8%) of our patients had nonglomerular ESRD, AD was found predominantly in those with glomerular ESRD: 46.5% of children with glomerular disease (20/43) compared to 18.5% (10/54) of children with nonglomerular disease had AD (p = 0.006). Specifically, focal segmental glomerulosclerosis (FSGS) tended to be more frequent in the AD group compared to the group without AD (33.3% vs. 14.9%, p = 0.056). 

Uncontrolled hypertension was more common in the AD group on univariate analysis. While children with AD had similar SBP and DBP when compared to those without AD, both SBP and DBP indices were significantly higher in the AD group. There were no significant differences between the AD and non-AD groups with regards to dialysis modality or other dialysis parameters (Table 1). 

### Multivariate analyses (Table 2) 

Model 1 of our logistic regression analysis shows that both BMI Z-score and the presence of glomerular disease are associated with AD. An increase of BMI Z-score by 1 is associated with a decrease in AD risk (OR = 0.52, 95% CI: 0.35 – 0.78). The presence of glomerular disease is a significant independent predictor of AD (OR = 4.58, 95% CI: 1.45 – 14.46). In contrast to the univariate analysis, the association between AD and SBP and DBP indices is insignificant in model 1. Logistic regression analysis using our model 2 shows similar results as shown in Table 2. 

**Figure 1:**
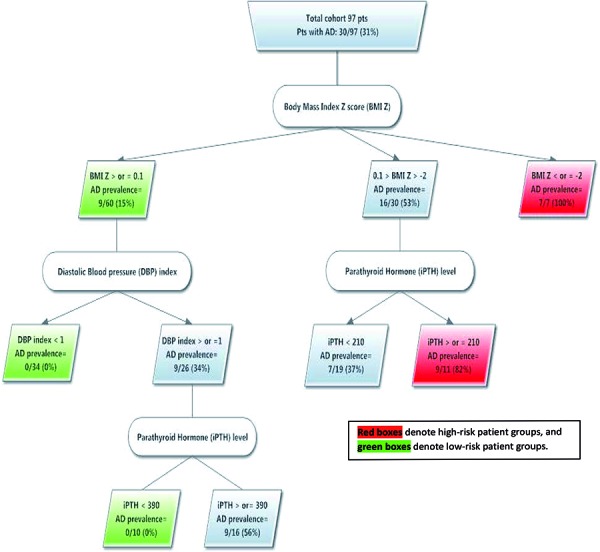
Nonparametric classification tree for aortic dilation (AD) demonstrating the high- and low-risk populations.

The results of the CART are shown in Figure 1. This model identifies three important clinical variables that can distinguish subpopulations at high versus low risk for AD: BMI Z-score, DBP index and intact parathyroid hormone (iPTH) level. As described before, 30 of 97 (31%) of our patients with ESRD had AD. CART classified two subgroups within this ESRD cohort that have an at least two-fold increased risk for AD (i.e. > 62%) and three low-risk subgroups at no more than half the overall risk for AD (i.e. < 15.5%). The high-risk subgroups include:1) patients with a BMI Z-score ≤ –2.0, with 7 of 7 (100%) patients having AD and 2) patients with a BMI Z-score in the range of –2.0 to +0.1 and an iPTH level ≥ 210 µg/dL, with 9 of 11 (82%) patients having AD. The low-risk subgroups include: 1) patients with a BMI Z-score > 0.1, with 9 of 60 (15%) patients affected, 2) patients with a BMI Z-score of ≥ 0.1 and a DBP index < 1 (where no AD was found among 34 patients), and 3) patients with a BMI Z-score of > 0.1, a DBP index ≥ 1 and an iPTH level< 390 µg/dL (where no AD was found among 10 patients). Among the whole cohort of 97 patients, the CART model could classify 62 (64%) patients as being at high or low risk for AD. For the patients classified as high- or low-risk, the CART model represents an efficient predictive tool with a positive predictive value of 89% (95% CI: 64 – 98%) and a negative predictive value of 100% (95% CI: 90 – 100%). 

## Discussion 

Survival in children with ESRD has increased over the last 20 years, but their standardized morality rate remains very high. CVD is the leading cause of death in adolescents and young adult patients with ESRD, and annual CV mortality rates are elevated several hundred fold in young adults with long-standing CKD [4, 15]. 

Left ventricular hypertrophy accelerated ischemic heart disease, premature dilated cardiomyopathy, aortic valve calcification, increased arterial stiffness, and arterial intima and media thickening are the most frequently observed CV alterations in young adult survivors of childhood-onset ESRD [16]. Vascular abnormalities in children develop in parallel with cardiac abnormalities early in the course of CKD and become more severe as ESRD is reached [17]. 

The literature describing the prevalence of and risk factors for thoracic AD in a young ESRD population is sparse [18]. Based on our findings, advanced kidney disease appears to represent a novel acquired etiology for thoracic aortopathy in children. 

AD is uncommon in healthy children and adolescents [13, 19, 20, 21], however, it occurs in 2.8% of children with hypertension [22] and in association with congenital abnormalities like bicuspid aortic valve [23] and syndromic or nonsyndromic genetic disorders like Turner, Ehlers-Danlos, Marfan, Alagille, and Beals syndromes [1, 24]. Children with ESRD represent a unique population in which multiple risk factors contributing to the development of aortopathy can coexist. These risk factors include volume overload, chronic anemia, anorexia, hypertension, and presence of arteriovenous fistulas. Furthermore, ESRD patients on chronic HD experience significant hemodynamic alterations because of abrupt changes in the intravascular volume and myocardial stunning [25, 26] secondary to frequent myocardial ischemia. These alterations might trigger a degenerative modeling of myocardium and vasculature. Thoracic aortopathy is often asymptomatic until an acute and catastrophic complication like dissection takes place [1, 20]. In the aforementioned conditions known to be associated with aortopathy, however, such complications may be seen at smaller aortic diameters than expected or even with normal aortic dimensions [1]. While it is presently unclear whether patients with ESRD and associated AD are categorically at increased risk for dissection, there are multiple case reports of aortic dissections in patients with autosomal dominant polycystic kidney disease [1, 13, 27] or cystinosis [28]. These reports raise at least some concern that patients with kidney disease may indeed have associated aortic pathology with potential for dissection. 

We found a 30.9% incidence of AD in our center’s ESRD population compared to reference incidences of 2.3% in healthy children and 2.8% in hypertensive children [22]. We also found that low BMI Z-score is the most influential risk factor for AD in all univariate, parametric multivariate, and nonparametric multivariate analyses. This relatively low BMI possibly reflects nutritional deficiencies, even though our patients with AD had mean BMI Z-scores still above the threshold used to define malnutrition, however, all malnourished children in our cohort had AD, and malnutrition is known to negatively impact CVD risk and mortality in both children and adults with CKD. Moreover, the inflammatory state associated with malnutrition is directly linked to a high risk of atherosclerosis known as the malnutrition–inflammation atherosclerosis complex [29, 30]. Our findings may therefore suggest that malnutrition with associated microinflammation and oxidative stress could also be an important determinant of aortopathy in pediatric and young adult ESRD. To date, we are not aware of clinical data showing such an association between malnutrition and vascular abnormalities. However, we believe that our observation of such an association between low BMI and AD may be valid, rather than merely reflecting the notion that thin persons tend to have larger aortas because we calculated Z scores using regression equations to determine where a specific aortic dimension lies in the normal distribution relative to BSA for that dimension. This approach in calculating Z scores adjusts for effects of BSA on the size of aorta, so that in normal subjects there is no residual relation between BSA and the size of aorta. Accordingly, lower BMI with subsequent lower BSA does not result in higher Z scores in aortic dimension unless there is an independent association. Any relationship of the Z scores with BMI should therefore independently reflect the impact of BMI on the size of the aorta. 

Our patients with AD were more likely to have glomerular disease and hypertension. At first glance, this association could be explained by the tendency of patients with glomerular disease to retain more fluid, leading to pre- and postcardiac overload. However, fluid-related variables, such as IDWG and normalized UF percentage, were not significantly higher in patients with AD compared to those without. This might suggest that both glomerulonephritis and AD share a common pathological pathway. Along these lines, Adedoyin et al. [31] also reported that other cardiac complications are relatively common in children with CKD secondary to glomerular disease, which may have immunological and inflammatory impact not only on the kidney but also the cardiovascular system. 

Furthermore, both the findings of Adedoyin et al. [31] and our findings may specifically implicate underlying FSGS as a risk factor for cardiovascular morbidity (cardiomyopathy and congestive heart failure in the former study and AD in ours). While our analysis only revealed a statistically insignificant trend for FSGS as a risk factor for AD, a definite conclusion cannot be made using our data because the number of patients with FSGS was relatively small. In addition to the possibility that these findings may be related to the fact that FSGS is the most common glomerular disease leading to ESRD at a young age, they also suggest that the immune or genetic mechanisms responsible for the development of FSGS may additionally negatively impact the cardiovascular system. 

Our data suggest that patients with AD are more likely to have uncontrolled or elevated BP than patients without AD in univariate analysis but not in multivariate analysis. Elevated BP has been a well-studied risk factor for AD in hypertensive adults [32, 33, 34] and children [22]. The most likely reason for hypertension not being a significant factor in multivariable analysis is the presence of more “powerful” predictors in our cohort, specifically malnutrition and the presence of glomerular diseases. In one study, the reported prevalence of AD in hypertensive children was 2.8% (0.5% higher than the reported prevalence of 2.3% in healthy children). Our cohort has an AD prevalence of 30.9%, indicating other factors more significant than elevated BP contributing to AD. Moreover, left ventricular hypertrophy markers, such as left ventricular mass indices, are similar between patients with and without AD in our study. 

Aortopathy, beginning with dilatation of the aorta and leading to aneurysm formation, is a subclinical condition that can be diagnosed only by using comprehensive aortic interrogation by noninvasive imaging strategies such as echocardiography. Such surveillance is likely not performed routinely in most centers. Our nonparametric CART is a highly predictive model based on clinical risk factors that are very likely available to physicians caring for ESRD patients and might be helpful in the identification of a high-risk population that will benefit from targeted close observation for developing AD. 

Despite of the increased awareness of the need to establish multidisciplinary care of adults and children with CKD [35], recent publications have highlighted the suboptimal quality of care for children with chronic illness [36] and for adults with CKD [37] or kidney transplant [38]. Similarly, patients in pediatric programs do not routinely receive the optimal quality of screening for cardiovascular complications such as routine echocardiograms [39]. This is one likely reason why our data are somewhat difficult to compare. Another reason is that, even at other pediatric centers where regular echocardiographic assessments are performed in the ESRD population, AD could be under-detected because measuring aortic dimensions is not part of the standard imaging protocol. As such, aortopathy may not be recognized unless the aortic root morphology is already severely abnormal and associated with aortic insufficiency. 

Our study has some significant limitations. First, this is an observational retrospective single center report. Second, our study describes a clinical observation, i.e., AD, that is not necessarily associated with a pathophysiologic process, i.e., aortopathy. Third, given the cross-sectional design of the study, longitudinal outcomes, and complications of AD cannot be evaluated. Lastly, gold-standard diagnostic measures, such as cardiac MRI and ambulatory blood pressure monitoring, were not available to further validate our data. Despite these limitations, we believe that our observations are significant enough to warrant further research into the occurrence of aortopathy in children with ESRD. Such further research could include prospective noninvasive surveillance with detailed assessment of the aortic root (as lack of such focus led to the exclusion of a number of echocardiograms from our retrospective, cross-sectional study) and concomitant measurements of inflammatory markers. These efforts could supplement and expand the findings presented here, especially because, to our knowledge, no prior research has evaluated the prevalence of AD among children with ESRD. Describing this prevalence represents the first significant step towards an improved understanding of this novel manifestation of CVD in young individuals with ESRD and thus at very high risk for CV morbidity and mortality. 

## Acknowledgment 

The first authors wish to acknowledge the International Society of Nephrology (ISN) as this work has been made possible through an ISN-funded fellowship for S.U. We also acknowledge grant support from the Center for Clinical and Translational Science and Training (UL1-RR026314). 

## Conflict of interest 

The authors declare no conflicting interest. No honorarium, grant, or other form of payment was given to any author to produce the manuscript. 

**Table 1.** Demographic characteristics by aortic dilation (AD) – univariate analysis (n = 97).

Variable	Patients with AD n = 30	Patients without AD n = 67	p-value
Demographics			
Age (years), Mean (SD)	12.3 (6.1)	11.68(6.8)	0.61
Sex (male), n (%)	18 (60.0%)	37 (55.2%)	0.63
Weight (kg), mean (SD)	48.8 (32.6)	39.6(19.7)	0.15
Height (cm), mean (SD)	141.1 (32.2)	134.5 (35.4)	0.39
BMI (kg/m^2^), mean (SD)	18.4 (3.7)	23.3 (7.15)	0.001
BMI Z-score	–0.7 ± 1.7	0.9 ± 1.3	< 0.0001
Malnourished patients, n (%)	7 (23.3%)	0 (0%)	N/A
Echocardiography			
Aortic root parameters (cm), mean (SD)			
Sinus level	2.7 (0.5)	2.25 (0.6)	0.0006
Sinus level Z-score	2.6 (0.7)	0.7 (0.8)	0.0001
Annulus	2.5 (2.8)	2.1 (2.8)	0.52
Annulus Z-score	0.5 (0.8)	–1.03 (0.6)	0.0001
Sinotubular junction	3.1 (4.2)	1.92 (0.5)	0.04
Sinotubular junction Z-score	2.3 (1.0)	0.18 (0.85)	0.0001
Ascending aorta	2.6 (0.5)	2.16 (0.6)	0.011
Ascending aorta Z-score	2.9 (1.2)	0.4(0.7)	0.0001
Other echocardiographic measures:			
Left ventricular mass (LVM)	114 (63.0)	96.1 (60.1)	0.2
Left ventricular mass index 2.7 m^2^	50.8 (28.3)	47.1 (22.9)	0.5
ESRD etiology			
Glomerular, n (%)	20 (66.6%)	23 (34.3%)	0.006
FSGS, n (%)	10 (33.3%)	10 (14.9%)	0.056
Blood pressure variables			
Uncontrolled hypertension, n (%)	26 (86.6%)	40 (59.7%)	0.01
SBP (mmHg), mean (SD)	131.4 (14.6)	130.3 (19.1)	0.80
DBP (mmHg), mean (SD)	84.8 (13.2)	82.1 (14.56)	0.46
BP index SBP > 1, n (%)	24 (80.0%)	37 (55.2%)	0.02
BP index DBP > 1, n (%)	22 (73.3%)	30 (44.7%)	0.009
Dialysis			
Duration (years), mean (SD)	1.2 (0.8)	1.6 (01.0)	0.44
Kt/V	1.8 (0.5)	1.9 (0.5)	0.42
Fluid Status			
Hemodialysis			
Normalized interdialytic weight gain	5.1 (2.3)	4.0 (1.6)	0.09
Normalized ultrafiltration	4.8 (3.6)	3.9 (1.7)	0.09
Normalized excess weight	1.6 (0.6)	1.5 (1.0)	0.6
Peritoneal dialysis			
Corrected ultrafiltration	666.6 (276.1)	689.0 (299.2)	0.85
Laboratory values			
Dyslipidemia, n (%)	21 (66.6%)	37 (55.2%)	0.32
Ca × P	60.5 (14.5)	59.3 (15.9)	0.68
iPTH (µg/dL), mean (SD)	422 (359)	375 (360)	0.56
Phosphorus (mg/dL), mean (SD)	6.5 (1.8)	6.5 (1.9)	0.67
Albumin (g/dL), mean (SD)	3.3 (0.6)	3.6 (0.7)	0.08
Hgb (g/dL), mean (SD)	11.5 (1.2)	11.3 (1.2)	0.44
Proteinuria, n (%)	21 (68.9%)	38 (56.70%)	0.40

BMI = body mass index; BP = blood pressure; Ca × P = calcium × phosphorus product; DBP = diastolic blood pressure; FSGS = focal segmental glomerulonephritis; Hgb = hemoglobin; iPTH = intact parathyroid hormone; SBP = systolic blood pressure.

**Table 2.** Multivariate logistic regression models to identify risk factors associated with aortic dilation.

Model 1*			Model 2**		
Clinical predictor of AD	Odds ratio (OR)	95% CI range	Clinical predictor of AD	Odds ratio (OR)	95% CI range
BMI Z-score	0.52	0.35 – 0.78	BMI Z-score	0.47	0.26 – 0.84
Presence of glomerular disease	4.58	1.45 – 14.46	Presence of glomerular disease	12.34	1.9 – 79
Weight (kg)	0.98	0.96 – 1.01	Weight (kg)	0.98	0.94 – 1.01
SBP index > 1	1.176	0.01 – 250	SBP index >1	1.08	0.1 – 413
DBP index > 1	1.35	0.02 – 118	DBP index >1	6.57	0.04 – 545
			Left ventricular mass index 2.7 m^2^	0.99	0.96- 1.02
			Presence of dyslipidemia	0.51	0.09 – 3.01
			Ca × P	0.20	0.04 – 1.5
			Hgb	0.86	0.48 – 1.55
			iPTH	1.00	0.99 – 1.01
			Albumin	0.32	0.10 – 1.03

BMI = body mass index; BP = blood pressure; Ca × P = calcium × phosphorus product; DBP = diastolic blood pressure; Hgb = hemoglobin; iPTH = parathyroid hormone; SBP = systolic blood pressure. *Model 1 includes the covariates of p-vales ≤ 0.15 in univariate analysis (Table 1). The hemodialysis parameters of normalized interdialytic weigh gain and normalized ultrafiltration were not included in the model because these values are available for less than 50% of the cohort (37/97 patients). **Model 2 includes the variables in model 1 in addition to other clinical variables that are known to be associated with cardiovascular and morbidity risk in ESRD population.
